# Protonic Conduction in the BaNdInO_4_ Structure Achieved by Acceptor Doping

**DOI:** 10.1021/acs.chemmater.0c04828

**Published:** 2021-03-10

**Authors:** Yu Zhou, Masahiro Shiraiwa, Masanori Nagao, Kotaro Fujii, Isao Tanaka, Masatomo Yashima, Laura Baque, Juan F. Basbus, Liliana V. Mogni, Stephen J. Skinner

**Affiliations:** † Department of Materials, Imperial College London, Exhibition Road, London SW7 2AZ, U.K.; ‡ Department of Chemistry, Tokyo Institute of Technology, 2-12-1-W4-17, O-okayama, Meguro-ku, Tokyo 152-8551, Japan; § Center for Crystal Science and Technology, University of Yamanashi, 7-32, Miyamae, Kofu, Yamanashi 400-0021, Japan; ∥ Department of Materials Characterization, Centro Atomico Bariloche (CAB), Av. Exequiel Bustillo, Bariloche Rio Negro 9500 8402, Argentina

## Abstract

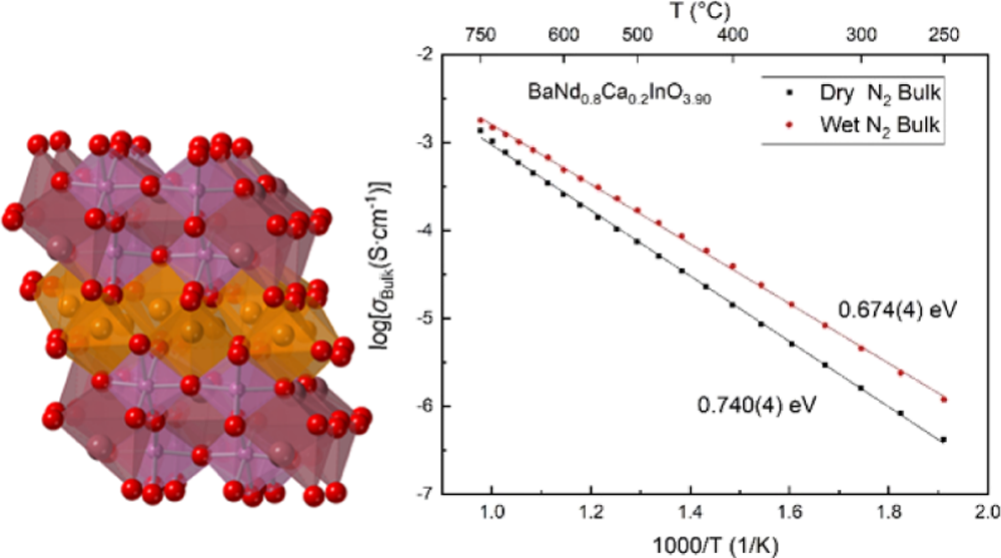

The potential of calcium-doped layered perovskite compounds, BaNd_1–*x*_Ca_*x*_InO_4–*x*/2_ (where *x* is the excess Ca content), as protonic conductors was experimentally investigated. The acceptor-doped ceramics exhibit improved total conductivities that were 1–2 orders of magnitude higher than those of the pristine material, BaNdInO_4_. The highest total conductivity of 2.6 × 10^–3^ S cm^–1^ was obtained in the BaNd_0.8_Ca_0.2_InO_3.90_ sample at a temperature of 750 °C in air. Electrochemical impedance spectroscopy measurements of the *x* = 0.1 and *x* = 0.2 substituted samples showed higher total conductivity under humid environments than those measured in a dry environment over a large temperature range (250–750 °C). At 500 °C, the total conductivity of the 20% substituted sample in humid air (∼3% H_2_O) was 1.3 × 10^–4^ S cm^–1^. The incorporation of water vapor decreased the activation energies of the bulk conductivity of the BaNd_0.8_Ca_0.2_InO_3.90_ sample from 0.755(2) to 0.678(2) eV in air. The saturated BaNd_0.8_Ca_0.2_InO_3.90_ sample contained 2.2 mol % protonic defects, which caused an expansion in the lattice according to the high-temperature X-ray diffraction data. Combining the studies of the impedance behavior with four-probe DC conductivity measurements obtained in humid air, which showed a decrease in the resistance of the *x* = 0.2 sample, we conclude that experimental evidence indicates that BaNd_1–*x*_Ca_*x*_InO_4–*x*/2_ is a fast proton conductor.

## Introduction

Acceptor-doped oxides with a wide variety of crystal structures have exhibited significant proton conductivity when measured under humid gas atmospheres.^1−7^ It has been proposed that these ceramic proton conductors can be applied to improve the performance of solid oxide fuel cell (SOFC) devices, developing the proton conducting ceramic fuel cell (PCCFC) device. SOFCs typically operate at high temperatures (600–1000 °C) as the ionic conductivity of the electrolytes reaches acceptable values in this range, depending on the composition.^2−12^ Proton conducting ceramics offer the advantage of fast ion conduction at lower temperatures, simplifying the device engineering.^13−16^ Among the structures that exhibit proton conduction, the acceptor-doped alkaline earth cerates and zirconates are most common and intensively investigated.^2,3,8−12,16^ To date, acceptor-doped BaCeO_3_ and BaZrO_3_ perovskite materials have been reported to show high proton conduction at intermediate-high temperatures (400–700 °C). According to the recent literature, a solid solution of BaCe_0.7_Zr_0.1_Y_0.2_O_3−δ_ yielded a total conductivity of 8 × 10^–3^ S cm^–1^ at 500 °C in humid N_2_.^16^ Although these perovskite (ABO_3_)-type oxides exhibited considerable proton conductivity, the poor tolerance to CO_2_ and rapid dehydration at elevated temperatures hampered the application of these compounds in the PCCFC devices.^9,11^

**Figure 1:**
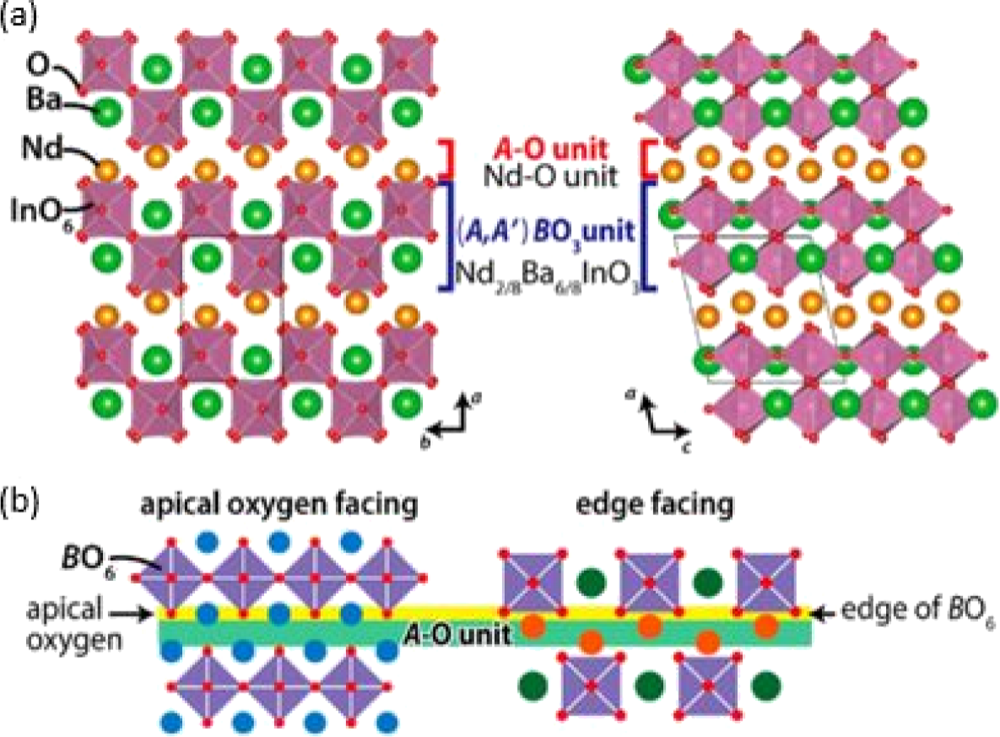
(a) Crystal structure of BaNdInO_4_ viewed along the *c* axis (left) and *b* axis (right). (b) Schematic diagrams of the apical oxygen facing mode of the rocksalt unit in a K_2_NiF_4_-type oxide (left) and the edge facing mode of the (Nd, Ba)_2_O_3_ unit (right) in a BaNdInO_4_. Reprinted with a permission from Fujii et al. (Chem. Mater. *26* 2488–2491). Copyright (2014) American Chemical Society.^17^

Reports of fast oxide ion transport in the novel layered perovskite structured material, BaNdInO_4_, prompted the investigation of proton incorporation and transport in this family of materials. This mixed oxide ionic and electronic conducting material, which was discovered by Fujii et al. in 2014,^17^ adopts a monoclinic crystal structure with the *P*2_1_/*c* symmetry. The crystal structure of BaNdInO_4_ consists of alternative stacking of rare earth oxide (Nd–O) units and perovskite (Ba_6/8_Nd_2/8_InO_3_) units with an edge-facing mode between the slabs as shown in Figure 1.^17,18^ Several cations such as strontium,^19^ barium,^20^ and calcium^21^ have been used to substitute the Nd site in order to create oxygen vacancies and therefore improve the electrochemical properties of these systems. Among all of these BaNdInO_4_-related oxides, BaNd_1–*x*_Ca_*x*_InO_4–*x*/2_ showed the highest oxide ion conductivities.^21^ However, there have been no reports of protonic transport within these acceptor-doped phases.

Therefore, in our present work, we have investigated the electrochemical properties and the proton conductivity of calcium-substituted BaNdInO_4_ through electrochemical impedance spectroscopy (EIS). The water uptake measurement along with the *in situ* DC conductivity measurement of the material was carried out using a combined system that couples an electrochemical measurement system with a thermogravimetric analysis (TGA) system.^22^ X-ray diffraction (XRD) analysis was conducted both before and after the sample hydration step, which confirmed an expansion in the lattice after the water incorporation. From these measurements, we conclusively demonstrate the fast proton transport of this family of oxides.

## Experimental Methods

### Synthesis

BaNd_1–*x*_Ca_*x*_InO_4–*x*/2_ (*x* = 0, 0.05, 0.10, 0.15, 0.20, 0.25, and 0.30) compounds were prepared by the solid-state reaction method. Stoichiometric amounts of Nd_2_O_3_ (99.9% purity, Alfa Aesar, pre-dried at 800 °C, 6 h), BaCO_3_ (99.999% purity, Sigma-Aldrich), In_2_O_3_ (99.9% purity, Alfa Aesar), and CaCO_3_ (99.99% purity, Sigma-Aldrich) were mixed and calcined at 1000 °C in static laboratory air for 14 h to decompose the carbonates and eliminate the CO_2_. The resulting ashes were then ball-milled (Planetary Micro Mill/300 rpm for 6 h) into a fine powder, pressed, and sintered at 1300 °C in air for 24 h with a heating/cooling rate of 5 °C per min to form the pellet-shaped samples. To obtain samples of high relative density, the ceramic pellet was crushed and ball-milled again into a fine powder, which then went through a uniaxial and isostatic pressing (∼290 MPa) process to form pellet samples. After being sintered at 1400 °C in air for another 24 h with a heating/cooling rate of 5 °C per min, pellets with 95–98% relative density were prepared. Samples are referred to in the following as BNC*xx*, where *xx* refers to the mol % Ca substitution. In this notation, the BaNd_0.80_Ca_0.20_InO_3.90_ is defined as BNC20.

### Characterization

The crystal structure of the formed phase in the BaNd_1–*x*_Ca_*x*_InO_4–*x*/2_ solid solution series was determined in static air by XRD using a PANalytical X’Pert PRO diffractometer (Cu Kα radiation). An Anton Parr HTK1200N oven with *z* axis adjustment was used to control the sample temperature to obtain high-temperature XRD (HT-XRD) patterns. The lattice parameters of the prepared samples were obtained through Le Bail refinement of the diffraction data using the FullProf software suite.^23^ The chemical composition analysis of the BaNd_1–*x*_Ca_*x*_InO_4–*x*/2_ compounds was performed by energy-dispersive X-ray spectroscopy with a JEOL 6010 LA scanning electron microscope.

To probe the electrochemical properties of the ceramic samples, EIS measurements were carried out with a Solartron Analytical 1260 frequency response analyzer (Solartron, UK) over the frequency range from 10^7^ to 10^–1^ Hz. The pellet samples were coated with platinum paste on the opposite faces and then annealed at 800 °C for 2 h in order to dry the platinum paste and ensure good adhesion between the sample surface and the platinum electrodes. Each sample was tested in a thermal cycle comprising a heating program with a heating rate of 5 °C/min to each temperature, starting from 250 °C, followed by 60 min thermal equilibration, with a step size of 25 °C until a maximum temperature of 750 °C was reached. This was followed by a cooling programme with the same steps and ramp rates to probe if there was any hysteresis in the material. The whole apparatus was sealed during the measurements, while the flow of compressed air or nitrogen was introduced through a drying tube containing CaSO_4_ before entering the impedance rig to create dry atmospheres. A water bubbler was connected into the system between the gas cylinder and the impedance rig to examine the influence of water vapors on the electrochemical properties of the materials. With this setup, it was possible to measure the same sample, first in the dry atmosphere and then in the wet atmosphere. Before the impedance spectroscopy measurement in the humid atmosphere, the sample was annealed in humid gas at 500 °C overnight to ensure that the equilibrium of the water incorporation was achieved. The impedance data were analyzed using ZView^24^ software package. We used the Zeiss Crossbeam 340 at Bariloche Atomic Centre to probe the topographic features of the sample surface before and after the wet annealing.

### Symmetrical TGA and DC Conductivity Measurement

TGA on the water uptake of the BNC20 sample under a humid atmosphere was conducted using the unique equipment developed by Caneiro et al.^22^ This equipment couples a symmetrical TGA system based on a Cahn 1000 electrobalance to an electrochemical system for *p*(O_2_) regulation. The internal environment of the apparatus was precisely controlled so that the TGA measurement had a sensitivity of 0.5 μg and noise of 5 μg peak to peak.^22^ The system measures two samples simultaneously. One of the two samples was held in an alumina crucible that was hung and attached to the electrobalance using a platinum wire for the TGA test. A DC conductivity measurement circuit was included in the system so that the electrical conductivity of a second sample with an identical geometry placed in exactly the same environment with the sample intended for the TGA could be measured simultaneously. The pellet sample used for the DC conductivity measurement was coated with platinum electrodes on both sides. A water bubbler was used to introduce water vapors into the system during the measurements.

## Results and Discussion

**Figure 2:**
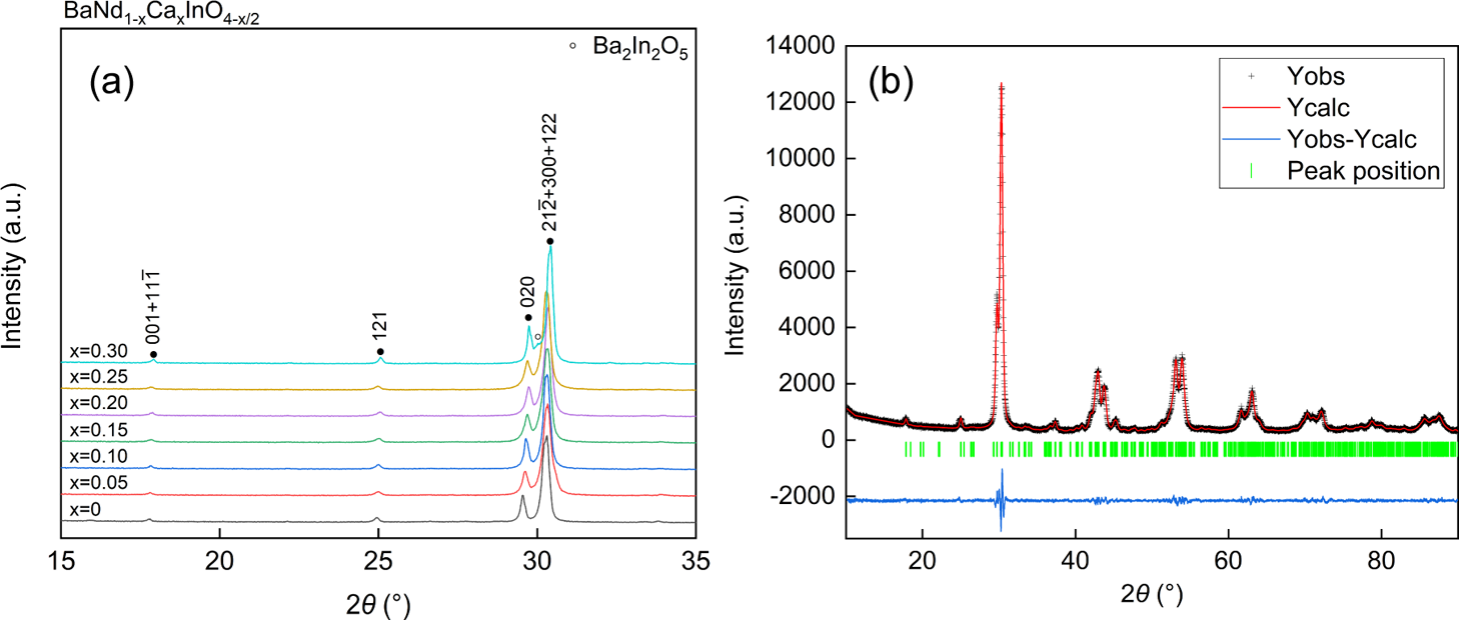
(a) Selected region (15–35°) of the measured XRD patterns of BaNd_1–*x*_Ca_*x*_InO_4–*x*/2_ (*x* = 0, 0.05, 0.10, 0.15, 0.20, 0.25, and 0.30) compounds and (b) Le Bail refinement result for the BNC20 composition.

XRD patterns of the as-sintered BaNd_1–*x*_Ca_*x*_InO_4–*x*/2_ (*x* = 0, 0.05, 0.10, 0.15, 0.20, 0.25, and 0.30) pellet samples, as shown in Figure 2, confirmed that a single phase of a monoclinic crystal structure with the *P*2_1_/*c* symmetry formed in all cases except for the 30% calcium-substituted sample. For the 30% substituted sample, all of the reflections in the pattern were indexed by a single monoclinic phase, while the appearance of an additional peak, as labeled with a hollow circle in Figure 2, suggested that a secondary phase of barium indium oxide had formed in this nominal composition sample.^25^

The substitution of calcium at the Nd sites in BaNd_1–*x*_Ca_*x*_InO_4–*x*/2_ caused a contraction in the unit cell volume as the calcium content *x* increased from 0 to 0.2. As listed in Table 1, the *b* lattice parameter showed a decreasing trend when the calcium content increased. The nonlinear fluctuation of the refined lattice parameters as the Ca content *x* exceeded 0.2 implied that the solubility limit of Ca in the system was approximately 25% (i.e. *x* = 0.25).

**Table 1:** Refined Lattice Parameters of the BaNd_1–*x*_Ca_*x*_InO_4–*x*/2_ (*x* = 0, 0.05, 0.10, 0.15, 0.20, 0.25, and 0.30) Compounds

composition	a (Å)	b (Å)	c (Å)	β (°)	V (Å^3^)
BaNdInO_4_	9.0931(1)	6.0443(1)	8.2585(1)	103.401(1)	441.54(1)
aBaNdInO_4_	9.0954(3)	6.04935(2)	8.25619(2)	103.4041(3)	441.89(1)
BaNd_0.95_Ca_0.05_InO_3.975_	9.0798(1)	6.0338(1)	8.2582(1)	103.414(1)	440.09(1)
BaNd_0.9_Ca_0.1_InO_3.95_	9.0930(1)	6.0296(1)	8.2643(1)	103.370(1)	440.83(1)
BaNd_0.85_Ca_0.15_InO_3.925_	9.0871(2)	6.0244(1)	8.2673(2)	103.291(2)	440.47(2)
BaNd_0.8_Ca_0.2_InO_3.9_	9.0644(2)	6.0120(1)	8.2627(2)	103.297(1)	438.21(1)
BaNd_0.75_Ca_0.25_InO_3.875_	9.1045(3)	6.0217(1)	8.2712(2)	103.294(3)	441.31(2)
BaNd_0.7_Ca_0.3_InO_3.85_	9.0941(2)	6.0248(1)	8.2594(1)	103.332(2)	440.34(1)

a The time-of-flight neutron diffraction data at 24 °C from ref (17).

**Figure 3:**
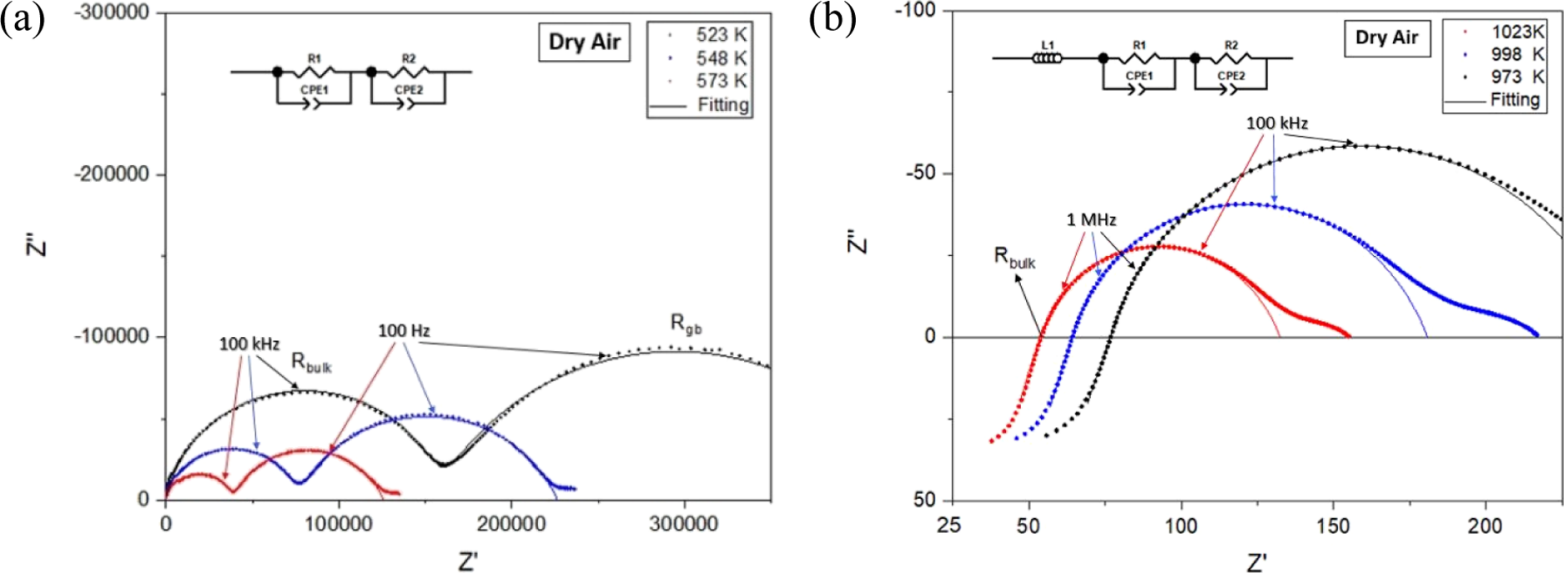
Impedance spectra of BNC20 in dry air at (a) low (523–573 K) and (b) high (973–1023 K) temperatures. Solid lines show the fit to the equivalent circuit models. Low frequency electrode response is omitted from the fit.

The electrical conductivity of the pristine BaNdInO_4_ together with the 10 and 20% calcium substituted samples was measured by EIS in dry air. No significant difference was observed between the data points obtained at the same temperatures from the heating and cooling cycles, as shown in the comparison between the total conductivities of the BNC20 sample measured on heating and cooling in wet air that are presented in Supporting Information as Figure S3. Representative fitted impedance spectra along with the equivalent circuits created to simulate the Nyquist plots are shown in Figure 3. As can be noticed, at low temperatures (523–573 K), the Nyquist plots contain two semicircles representing the bulk and grain boundary contributions, respectively, which can be simulated with two R-CPE (constant phase element) components connected in series. The associated capacitances, *C*_1_ and *C*_2_, can be estimated using the relation 2π*f*_max_*RC* = 1 and were ∼1.1 × 10^–11^ and ∼1 × 10^–8^ F cm^–1^, indicative of the bulk and grain boundary responses, respectively, where *f*_ma*x*_ is the frequency at the arc maximum. However, at high temperatures, the bulk resistance can only be obtained as the left intercept of the plots with the real part of the impedance (*Z*′), and the complex impedance plots can be simulated with one inductor and two R-CPE components connected in series.

**Figure 4:**
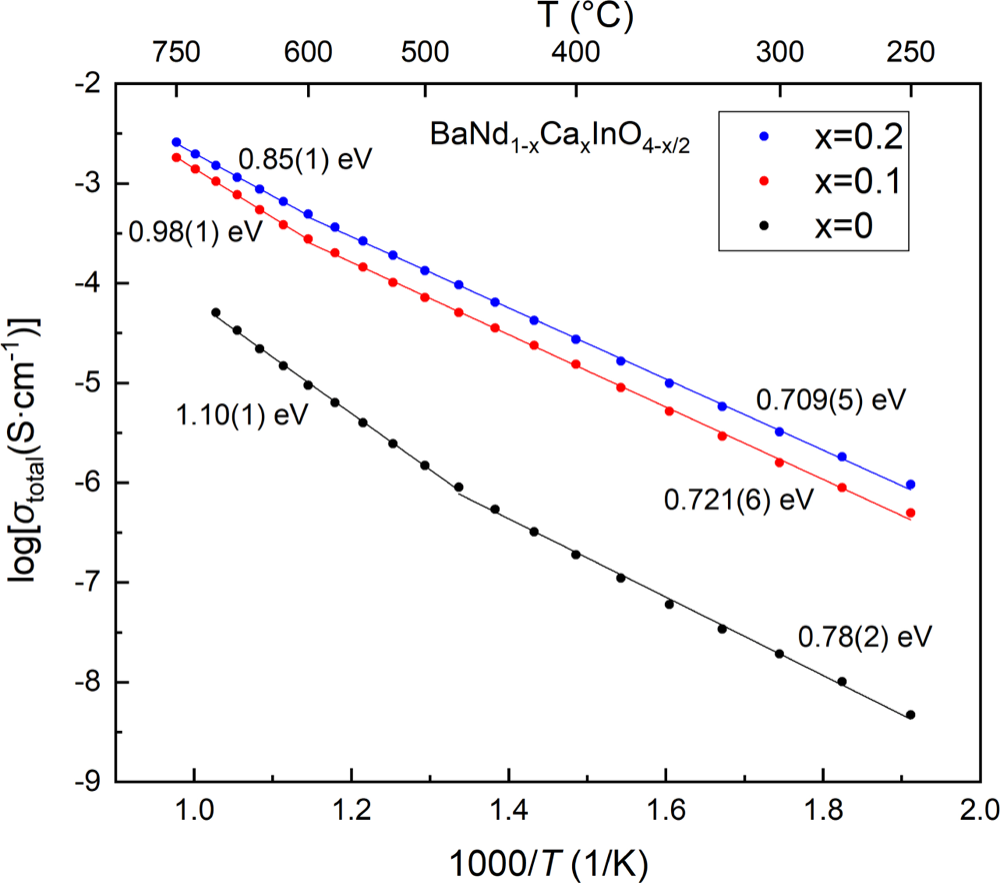
Arrhenius plots of the total conductivity of the BaNdInO_4_ (black), BNC10 (red), and BNC20 (blue) samples measured in dry air with the calculated activation energies displayed in both low and high temperature regimes.

The total conductivities were calculated using the addition of bulk and grain boundary resistance and the geometrical parameters of the pellets. All the three Arrhenius plots of the total conductivities are presented in Figure 4, which indicate a significant improvement of the total electrical conductivity on calcium substitution at the Nd^3+^ site. Among those samples, the BNC20 sample showed the highest total conductivity of 2.6 × 10^–3^ S cm^–1^ at 750 °C, while the total conductivity of the BNC10 sample was 1.8 × 10^–3^ S cm^–1^ at the same temperature. According to the EIS results, the electrical conductivity of the BaNdInO_4_ pristine material could be enhanced by 1–2 orders of magnitude over a large temperature range through calcium substitution. This enhancement can be ascribed to the formation of oxygen vacancies in the system by acceptor doping
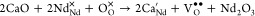


A change in the slope of the Arrhenius plots is observed in all three chemical compositions, which is not due to any irreversible chemical change during the impedance measurements, as the data obtained on a cooling cycle for all samples showed no significant difference compared to those obtained on the heating cycle. One hypothesis is that the change in the activation energies is due to the change of the dominant charge carriers in the system. In the low temperature range (250–500 °C for the parent material and 250–600 °C for the Ca-substituted samples), the materials exhibit mixed conduction (electronic and ionic), which allows for a mixture of charge carriers to flow through the system including oxide-ion and hole conduction.^17,21^ However, when the temperature was increased (>500 °C for the parent material and >600 °C for the substituted samples), oxide ions became the dominant charge carriers, as the number of hole defects was reduced due to the loss of oxygen in the system according to the defect equation below
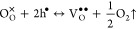


As a result of this reduction in p-type transport, the activation energy of the pristine material increases from 0.78(2) eV (mixed p-type and oxide-ion conduction) to 1.10(1) eV (predominant oxide-ion conduction) as the temperature increases. As for the calcium-doped samples, the slope change temperature is higher because of the higher concentration of oxygen vacancies in the system due to the acceptor doping, which inhibits the loss of oxygen to some extent, according to the equation above. Therefore, the activation energies of the BNC10 and BNC20 samples changed from 0.721(6) and 0.709(5) eV, respectively, to 0.98(1) and 0.85(1) eV when the temperature increased beyond 600 °C.

The Arrhenius plots also indicate that the activation energy of the pristine material can be reduced by 10 and 20% Ca doping in both the lower and higher temperature ranges, which could also be due to the increase in oxygen vacancies.

To probe the potential for protonic conductivity, water vapor was introduced into a sealed tube in the EIS system by placing a water bubbler in line between the supply gas cylinder and measurement apparatus. The flowing gas carried ∼3% H_2_O (25 °C) vapor into the system, which was then incorporated into the BNC20 and BNC10 samples forming protonic defects, as shown in the equation below
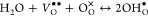


**Figure 5:**
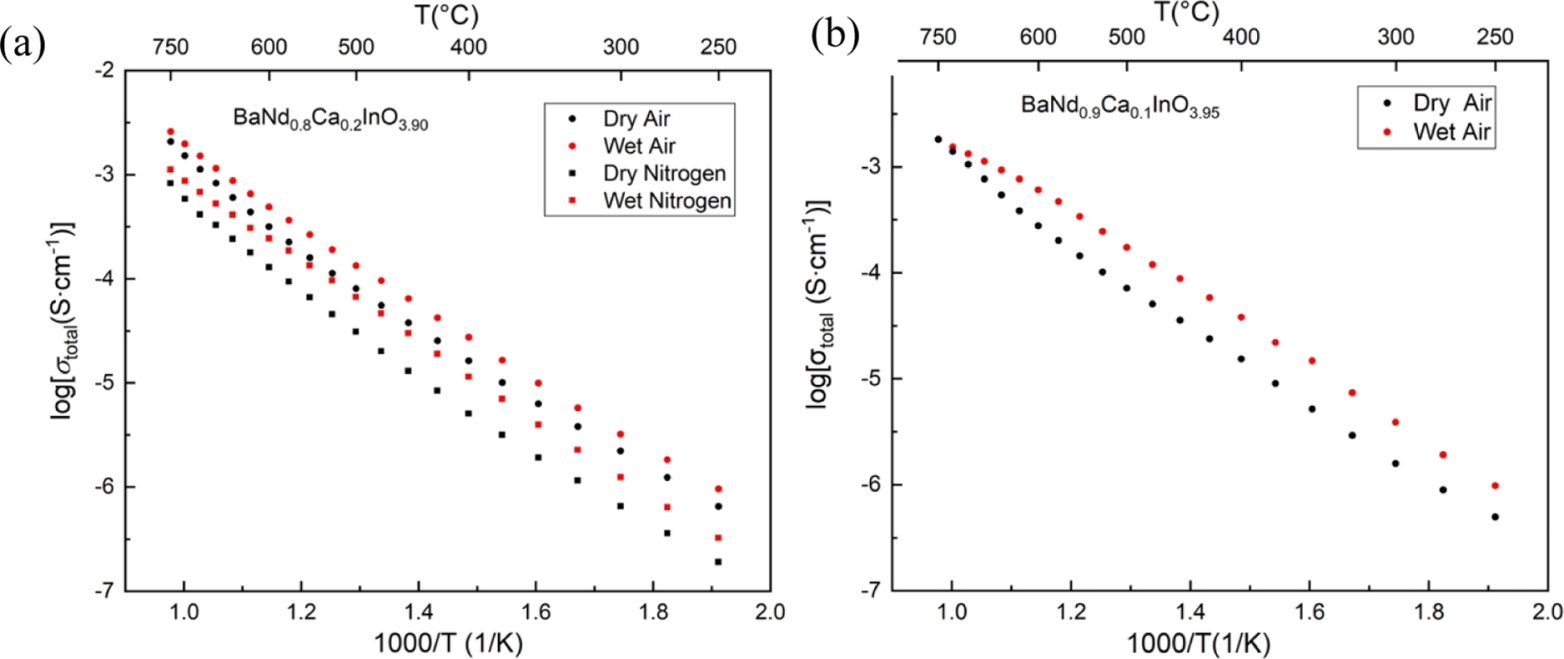
Arrhenius plots of the total conductivity of the (a) BNC20 and (b) BNC10 samples measured in dry and wet atmospheres.

Each sample was annealed in the humid environment at 500 °C overnight to ensure that the equilibrium of the water incorporation was achieved before the “wet” impedance spectroscopy measurement was performed. As can be seen in Figure 5, both BNC10 and BNC20 samples exhibited higher total conductivity under humid air than those obtained in dry air over a large temperature range (250–750 °C), which could be ascribed to the formation of protonic defects in the material. The total conductivity of BNC20 in wet air at 500 °C was 1.3 × 10^–4^ S cm^–1^, which was higher than the total conductivity of most of the state-of-the-art proton-conducting materials measured under the same conditions.^1^ EIS measurements on the BNC20 sample were also carried out in dry and wet N_2_ atmospheres, resulting in a similar trend as observed in dry air. The total conductivity of the BNC20 sample measured in a N_2_ atmosphere was lower than that when measured in air, which implies that this material exhibits p-type conduction in O_2_-rich atmospheres. This result is consistent with the previous reports.^19,21^

**Figure 6:**
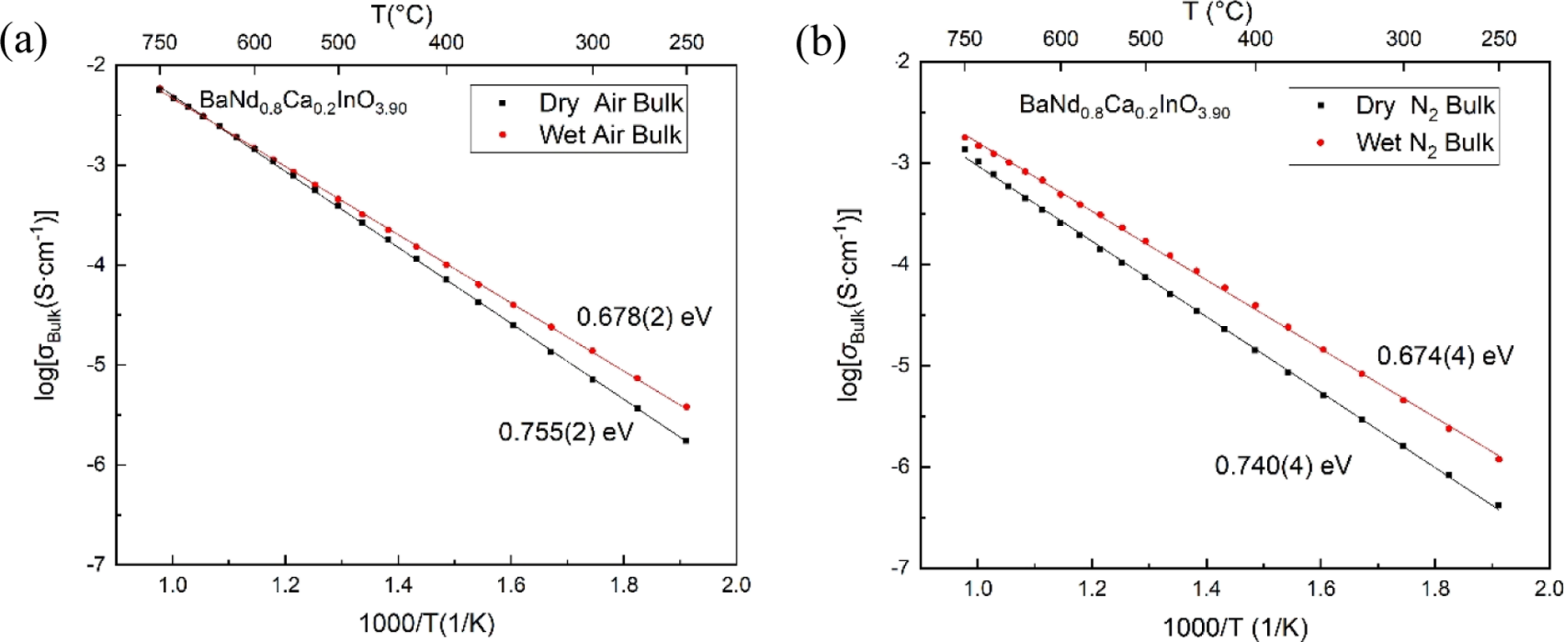
Arrhenius plots of bulk conductivity of a BNC20 sample under (a) dry and wet air and (b) dry and wet N_2_, respectively.

In order to investigate the influence of humidity on the electrochemical properties of BNC20 samples more deeply, the bulk conductivity was deduced by extracting the bulk resistance and calculating according to the brick layer model,^26^ assuming that the area of the bulk in the material equals the whole area of the sample. As shown in Figure 6, four Arrhenius plots of the bulk conductivity of BNC20 samples under different atmospheres fit well with a straight line, which gives the activation energies for bulk conductivity as 0.755(2) eV in dry air, 0.678(2) eV in wet air, 0.740(4) eV in dry N_2_, and 0.674(4) eV in wet N_2_. There was an increase in the bulk conductivities of the samples both in air and in N_2_ when water vapor was introduced to the system. The influence of the humidity on bulk conductivities was more significant in a N_2_ atmosphere than that in air, which can be ascribed to the excessive amount of oxygen vacancies in an O_2_-deficient environment according to the equation below
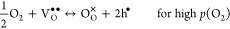


In addition, the activation energies for the bulk conduction decreased on introducing water vapor to the system, which implies a change in the conduction mechanism in the bulk material. As the proton mobility is higher than that of oxygen ions,^15,27^ the decrease in the activation energies could be evidence of proton conduction in the bulk material. If we compare the data in Figure 6, the activation energy of the bulk conductivity in both dry and wet atmospheres is independent of *p*(O_2_) within the O_2_–N_2_*p*(O_2_) range, suggesting limited impact of electronic charge carriers on the activation energies. In conclusion, the BNC20 sample exhibits p-type and oxide-ion mixed conductions under dry atmospheres within the O_2_–N_2_*p*(O_2_) range, while the incorporation of humidity can significantly increase the conductivity in the bulk and decrease the activation energies of the bulk conductivity, which implies that this material may exhibit triple (oxygen-ions, protons, and holes) conduction under wet atmospheres. The transport number of protons, *t*_p_, can be estimated using the equation
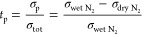
assuming that in dry N_2_, the total electrical conductivity is attributed to oxide ions alone, and thus, the proton conductivity could be estimated by the subtraction of total conductivity in wet N_2_ and the total conductivity in dry N_2_. In the temperature range from 250 to 475 °C, the transport number of protons *t*_p_ increased from 0.41 to 0.57. Then, a decreasing trend with further increases in temperature was observed, which is likely due to the dehydration of the sample at high temperatures. The transport number as a function of temperature is illustrated in Figure S4, which is included in the Supporting Information. Further direct measurements would be required to verify the assumption and the transport number of protons in this material.

**Figure 7:**
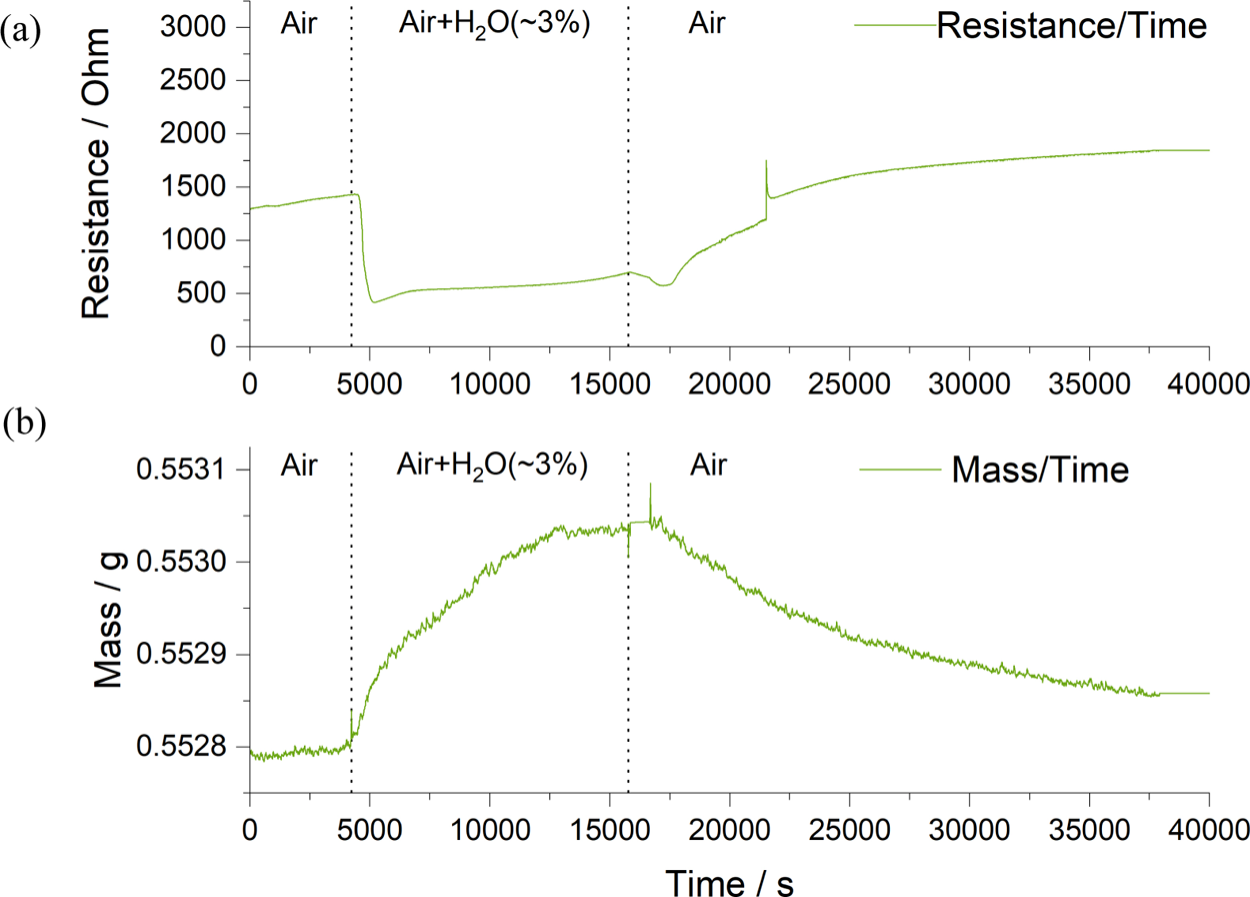
(a) Resistance and (b) mass vs time in the coupled TGA and conductivity measurements recorded at 500 °C.

The simultaneous TGA and four-probe DC conductivity measurement were conducted using a coupled apparatus. Two BNC20 samples were placed in exactly the same chemical environment in an airtight quartz tube which was heated to 500 °C. One sample was placed in an alumina crucible hanging from one of the arms of the symmetrical electrobalance, while the other sample was coated on both faces with platinum paste and located just below the crucible on a blocking tube for the conductivity measurement. Figure 7 shows the mass and resistance change of the BNC20 sample as the atmosphere changed from dry to wet and from wet to dry after reaching a plateau in both resistance and mass, which can be considered as a sign of full hydration in these two samples.

As depicted in Figure 7a, a few minutes after the introduction of water vapor, a dramatic decrease in resistance was observed, while the process of water uptake in mass was much slower, taking over 3 h to reach a plateau. However, the increase in mass, as shown in Figure 7b, started immediately after the change of atmosphere, while the decrease in sample resistance seemed to have a “delay” of 240 s. The difference in the behavior of mass and resistance change of the sample could be ascribed to the enhancement of water uptake in the BNC20 sample by platinum coating on the surface, or application of voltage upon the sample, or the sudden change in the conducting mechanism as the sample for conductivity measurement absorbed water to some extent. After full hydration, the chemical composition of the sample was calculated to be BaNd_0.8_Ca_0.2_InO_3.90_·0.011H_2_O. The maximum concentration of protonic defects **OH**_**O**_^**•**^ in the BNC20 sample at 500 °C was calculated to be 2.2 mol %. In comparison, the recovery process both in mass and resistance after switching to a dry atmosphere was much slower. TGA together with the DC conductivity measurement agrees well with the results obtained from the earlier EIS analysis, which again indicates that calcium-substituted BaNdInO_4_ is a protonic conductor under a humid atmosphere.

**Figure 8:**
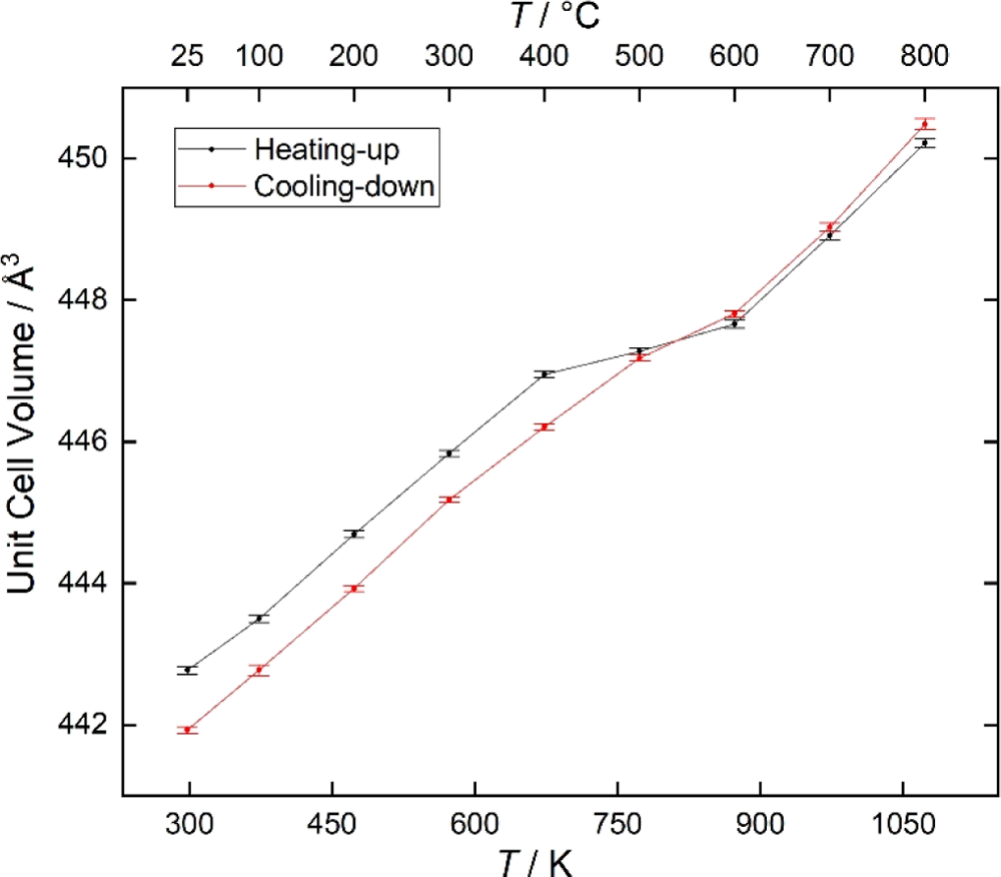
Fitted unit cell volume of the hydrated BNC20 sample versus temperature.

**Figure 9:**
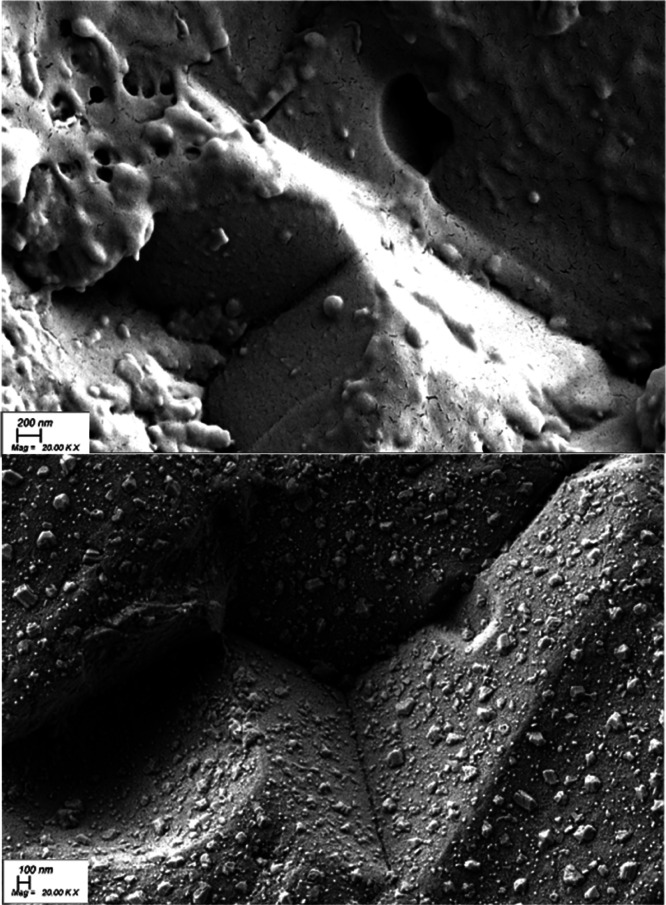
Secondary electron microscopy images of the BNC20 sample before (top) and after (bottom) being annealed at 500 °C in a humid environment for a week.

After the TGA measurement described above, the BNC20 sample was hydrated again at 500 °C in humid air for 48 h in preparation for the HT-XRD measurements. The hydrated sample was heated at a rate of 5 °C/min from 100 to 800 °C with a 100 °C step interval after an initial measurement at room temperature. After being heated to 800 °C for 1 h, the dehydrated sample was examined again on cooling in order to make a comparison with the previous data. All HT-XRD patterns were refined using the FullProf software suite^23^ through the Le Bail method. A clear lattice expansion was observed in the fitted unit cell volume of the hydrated BNC20 sample on heating compared with the data obtained on cooling, as shown in Figure 8, which is further evidence to suggest that BaNd_1–*x*_Ca_*x*_InO_4–*x*/2_ is a proton conductor under a humid atmosphere. The difference between the unit cell volume of the dehydrated sample obtained here at room temperature (441.93(2) Å^3^) and the one obtained in the previous sample (438.21(1) Å^3^) could be ascribed to the *z*-shift in different sample holders. The degree of hydration of the sample and the chemical change of the material after being annealed in wet atmosphere for a long period of time may also lead to a change in the lattice parameters obtained in the XRD measurements. Some evidence including the conductivity measurement results and the XRD results of the sample being annealed in wet atmosphere have suggested that the BaNd_1–*x*_Ca_*x*_InO_4–*x*/2_ experienced an A-cation exsolution forming Ba(OH)_2_/BaCO_3_ on the surface. Direct evidence showing this A-cation exsolution was observed in the SEM image taken from the sample, which had been annealed at 500 °C in the humid environment for a week. As can be seen in Figure 9, after wet annealing, a great number of faceted grains grew out from the original grain, which could be identified as BaCO_3_ using XRD.

## Conclusions

In summary, calcium substitution at the Nd site can significantly increase the total conductivity of BaNdInO_4_ pristine material by 1–2 orders of magnitude in dry air. Under dry atmosphere, the highest total conductivity of 2.6 × 10^–3^ S cm^–1^ was obtained in the BaNd_0.8_Ca_0.2_InO_3.90_ sample at 750 °C in air. Both the total conductivity, σ_Total_ (S cm^–1^), and the bulk conductivity, σ_Bulk_ (S cm^–1^), of the BNC20 sample were enhanced in wet atmosphere over a large temperature range (250–750 °C) compared with those measured in dry atmosphere. The incorporation of water vapor was found to lower the activation energies of the bulk conductivity of the BNC20 sample from 0.755(2) to 0.678(2) eV in air, which suggests proton conduction in the system. TGA and the simultaneous DC conductivity measurement showed a consistent result as the mass increased and the resistance of the sample decreased when the water vapor was introduced. The protonic defect concentration of the saturated BNC20 sample was 2.2 mol % according to the TGA measurement. Apart from that, a thermal expansion of the BNC20 sample after hydration was observed under HT-XRD measurements. Several experimental results have indicated that calcium-substituted BaNdInO_4_ oxides can be used as a proton conductor. However, an A-cation exsolution process was observed in the BNC20 sample, which was annealed at 500 °C in a humid atmosphere over a week, which suggested that this type of material shows poor chemical stability when being exposed to the humid environment for a long period of time.

## Supporting Information Available

Full XRD patterns for the entire solid solution series with 0 < *x* < 0.3, *in situ* XRD patterns for the BNC20 sample (*x* = 0.2) recorded between 25 and 800 °C on both heating and cooling, conductivity data recorded on both heating and cooling cycles, calculated proton transport number as a function of temperature, and secondary ion mass spectrometry data showing the incorporation of deuterons under wet exchange conditions (PDF)
