# Supplementary material for: Protonic Conduction in the BaNdInO4 Structure Achieved by Acceptor Doping
Source: Chem Mater. 2021 Mar 10;33(6):2139–46. doi: 10.1021/acs.chemmater.0c04828 (PMC8042909; doi:10.1021/acs.chemmater.0c04828)
Supplement: Supplementary file 1 — cm0c04828_si_001.pdf [file cm0c04828_si_001.pdf]

Electronic Supplementary Information

Protonic conduction in $BaNdInO_4$ Structure achieved by acceptor doping

Yu Zhou[(a)], Masahiro Shiraiwa[(b)], Masanori Nagao[(c)], Kotaro Fujii[(b)], Isao Tanaka[(c)], Masatomo Yashima[(b)], Laura Baque[(d)], Juan F. Basbus[(d)], Liliana V. Mogni[(d)] and Stephen J. Skinner[(a)*]

(a) Department of Materials, Imperial College London, Exhibition Road, SW7 2AZ, London, UK

(b) Tokyo Institute of Technology, Department of Chemistry, 2-12-1-W4-17, O-okayama, Meguro-ku, Tokyo 152-8551, Japan

(c) Center for Crystal Science and Technology, University of Yamanashi, 7-32, Miyamae, Kofu, Yamanashi 400-0021, Japan

(d) Centro Atomico Bariloche (CAB), Department of Materials Characterization, Av. Exequiel Bustillo 9500 8402 Bariloche Rio Negro, Argentina

* s.skinner@imperial.ac.uk

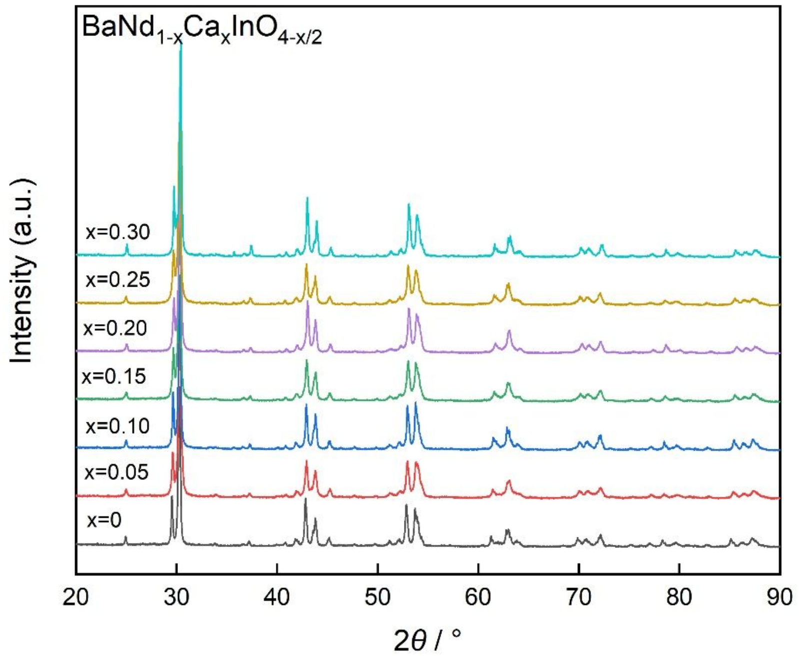

Figure S1. Full XRD patterns of $BaNd_{1-x}Ca_xInO_{4-x/2}$ compounds.

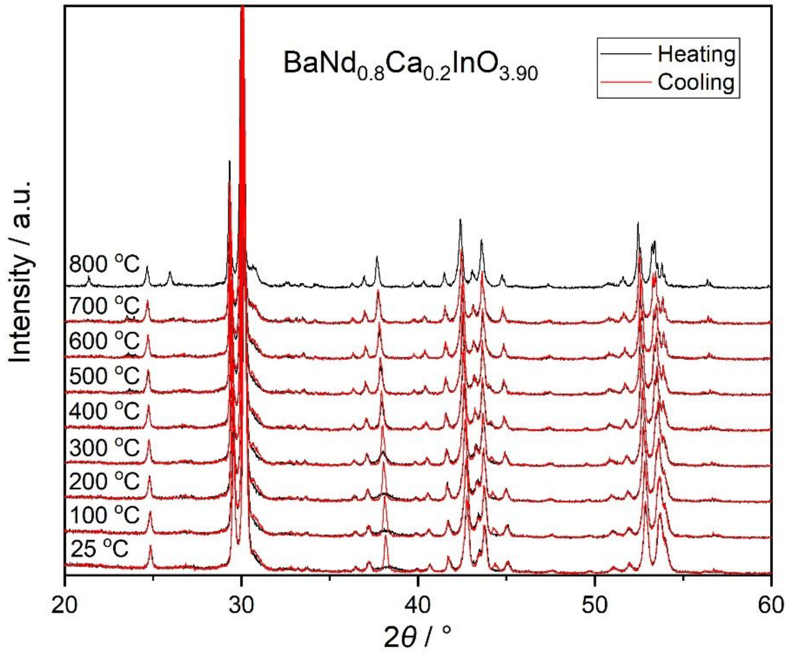

Figure S2. High temperature X-ray diffraction patterns of the hydrated BNC20 sample on heating (black) and cooling (red) cycles. Note the appearance of additional reflections at ~22 and ~27° 2θ at 800 °C that disappears on cooling. This indicates a potential phase transition that is currently undetermined. A further peak evident at 44° 2θ on cooling is of unknown origin.

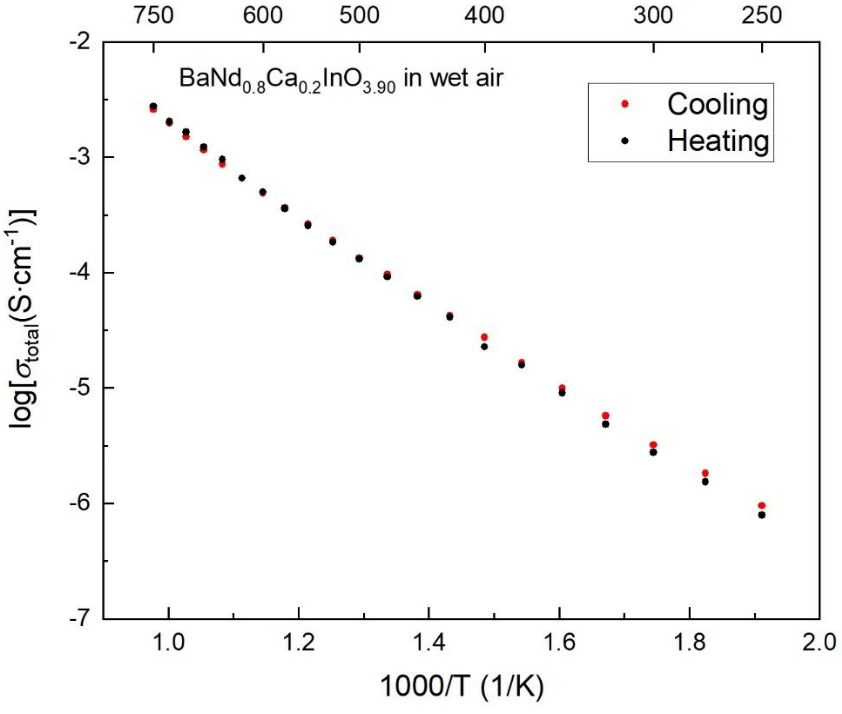

Figure S3. Arrhenius plots of the total conductivity of the BNC20 sample measured in heating (dark circles) and cooling (red circles) cycles in wet air.

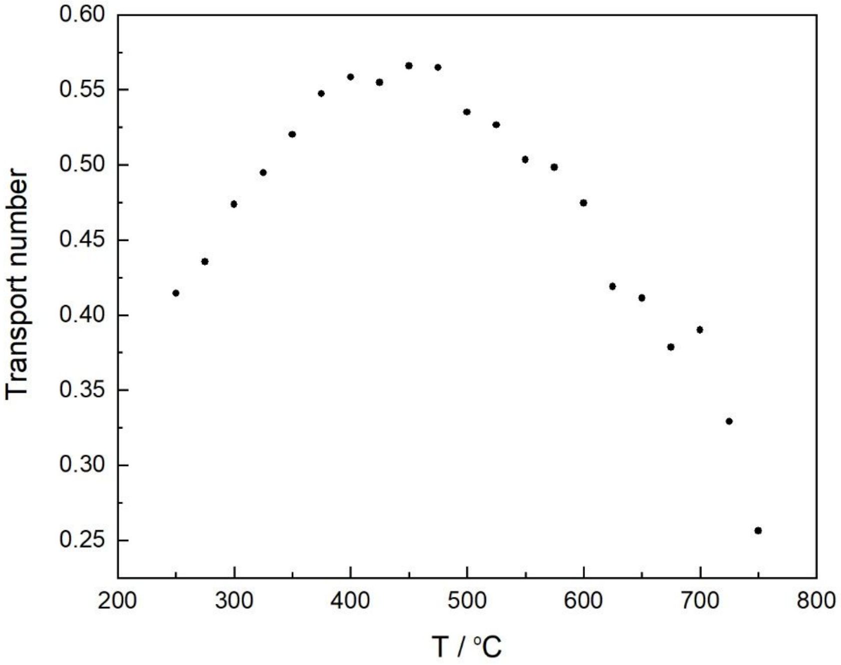

Figure S4. The transport number t_p as a function of temperature derived from the wet conductivity data of the BNC20 sample measured in wet N_2.

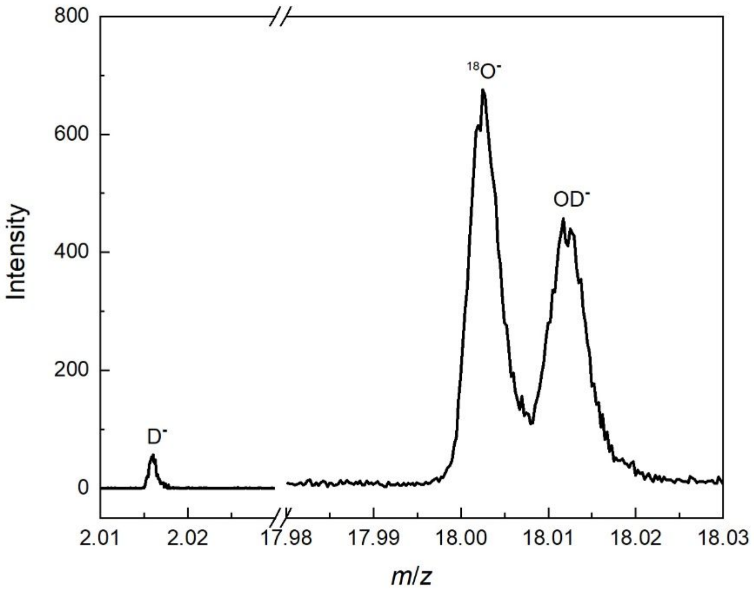

Figure S5. Selected region of the Secondary Ion Mass Spectrum of the D_2O exchanged BNC20 showing the presence of both OD^- and D^- species, confirming the incorporation of these species in the ceramics.

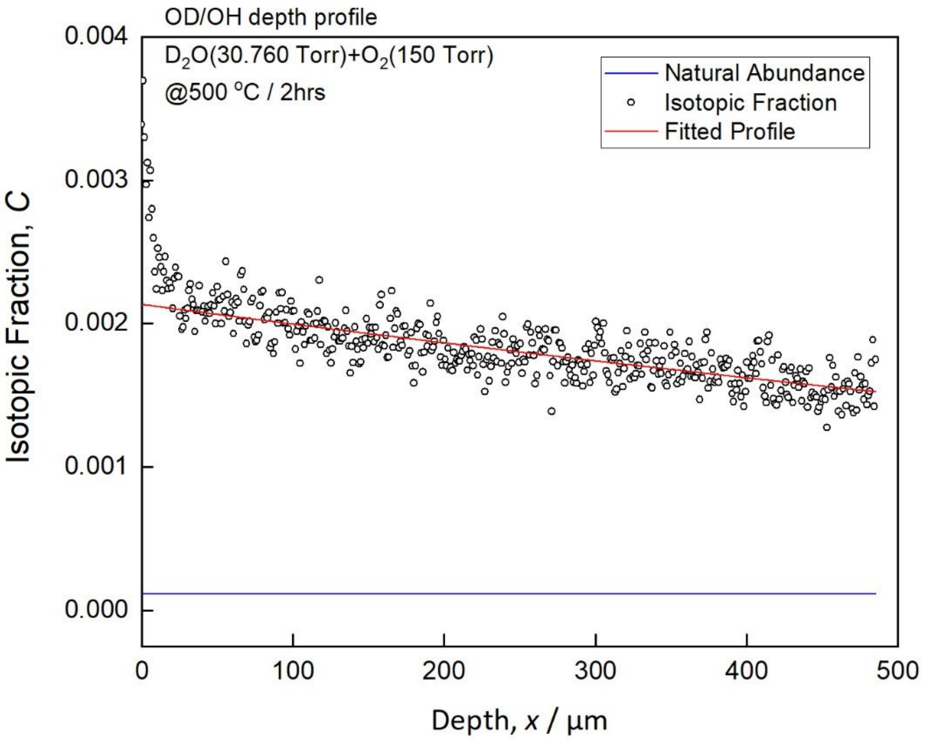

Fig S6. OD/OH depth profile of the BNC20 sample obtained by SIMS after being exchanged in the D_2O (30.760 Torr) + O_2 (150 Torr) atmosphere at 500℃ for 2 hours.

Experimental Details.

In the wet Isotope Exchange Depth Profiling (IEDP) experiments, a sealed tank storing D_2O was immersed in a water bath to create a wet atmosphere containing D_2O water vapour (~30 Torr) and oxygen (150 Torr). The samples were annealed at certain conditions to introduce the deuterium tracer creating a D/H profile which then can be recorded using the secondary ion mass spectrometry (SIMS) technique. The mass spectrum in Fig S5 shows the deuterium peak and the OD peak in comparison to the ^{18}O peak which yields much higher deuterium isotope fraction than the natural abundance of D as can be seen in the 500℃ D_2O exchanged depth profile. Fig S6 provides further evidence for the existence of protonic conduction in the BNC20 samples.
